# Hospital Meetings

**Published:** 1896-04-25

**Authors:** 


### HOSPITAL MEETINGS.

### HOSPITAL FOR EPILEPSY AND PARALYSIS.

That the interest taken by the subscribers and the public generally in this hospital requires rousing was clearly shown by the attendance at the annual meeting, which was held at the hospital, Portland Terrace, Regent's Park, N.W., on the 21st inst., under the presidency of the Earl of Carnwath. Only about a dozen persons, exclusive of the staff of the hospital, put in an appearance. Seeing that the hospital, though containing but 25 beds, treats a class of disease to which the human race in the present day is especially liable, and seeing that, in addition to this, it is one of the two or three which at present exist in London specially for the treatment of nervous disorders, this hospital deserves more support than it now gets. The Chairman, in moving the adoption of the report and accounts, mentioned that he had issued an appeal for funds on being asked to preside at that meeting, and as a consequence the funds of the hospital had been augmented by some £50. The report for the year 1895, although most satisfactory as regarded the work done in the hospital, was not satisfactory as regarded the contributions to the hospital funds; in fact, during the last few years there had been a decided decrease in the revenue of the hospital. The deficit for the year had to be supplied by drawing on the small capital for about £300, this following on a similar draft in 1894 of £200. Fewer in-patients were treated in 1895 than in previous years, *i.e.*, 106 against 118, 114, and 116 in the three preceding years, but the average length of residence had increased, viz., 77 days in 1895, against 72 in 1894, 65 in 1893, and 60 in 1892.

### HOME HOSPITALS' ASSOCIATION.

The annual meeting of the Home Hospitals' Association was held at Fitzroy-square, W., on the 21st inst., Mr. W. H. Bennett in the chair.

The Chairman, in moving the adoption of the report, said it was a matter for congratulation that, in these days of competition, the popularity of Fitzroy House was not only maintained, but was increasing. They had had to reject more patients during the past year than at any previous time, and there had also been a large addition in the number of medical practitioners who sent patients to the Home Hospital. In order to dispel some doubts which still seemed to exist in certain quarters, he explained that the Home Hospitals' Association was a home—a home in sickness—rather than a hospital, an institution where the patient found the same sort of food, the same attendance, and all the comforts which he could expect in his own house, together with the same facilities for seeing his friends, subject only to the restrictions laid down by his medical man.

Mr. Christopher Heath, F.R.C.S., seconded.

Mr. Henry C. Burdett said the strongest testimony to the success of Fitzroy House was the fact that so many eminent members of the medical profession had come there that day to support them. The reason for their support was that the more they knew of Fitzroy House the more they valued and liked it. In his opinion Fitzroy House had become as essential in its way to the advancement of surgery in private medical practice in London as anything which could be mentioned. He said that, because despite the very numerous and influentially-supported homes which had sprung into being since the foundation of the Home Hospitals' Association, this association had practically, without advertisement, certainly without effort, relying mainly upon the soundness of the work done within its walls, and the excellence of its arrangements, continued to maintain and earn for itself the gratitude and affection not only of its original founders, but of the members of the medical profession, and that numerous and ever-extending number of the general public who had been treated at Fitzroy House. He was pleased to see that last year the amount of the income was larger than it had ever been before, and that at the same time the expenditure showed a reduction when compared with that of the previous year.

The report was adopted. The other business was mainly formal, except that it bore testimony to the devotion of the Treasurer (Mr. Cox), who for the last eighteen years has attended regularly at least once a month to audit the accounts and supervise the expenditure; to Mr. T. Almond Hind, the able honorary secretary; and to Miss Pearson (the lady superintendent) and her staff of nurses, whose skill and efficiency were testified to by every one of the representative members of the medical profession who took part in the proceedings.

### WEST HAM HOSPITAL.

The festival dinner of the West Ham Hospital took place at the Holborn Restaurant on the 15th inst., Mr. A. F. Hills in the chair. Most of the after-dinner speakers urged that the chief necessity of the hospital at the present time was annual subscribers. Only about £760 was received last year from this source to meet an ordinary expenditure of £3,900, an expenditure which will be considerably increased in the future, in order to keep up the recently opened new wing which has been added through the munificence of Mr. Passmore Edwards and others. This hospital is situated in what is essentially a manufacturing district, and Mr. Passmore Edwards, in the course of his reply to the toast, "Prosperity to the Hospital," pointed out that, in his opinion, the large capitalists who made their money out of the borough did not support the hospital as liberally as they should. If the school children could during the year raise about £300 in pence as a contribution to the fund of the hospital, surely the large capitalists could do better than they were doing at the present time. Mr. E. Rider Cook dissented from this view on the ground that the manufacturers were already overburdened with local rates, and held that, as the district was filled with working-men who worked in other parts of London, but whose children were educated at the expense of the local rates, therefore it was not only from West Ham that contributions should come but from all parts of the metropolis. The contributions announced as a result of the dinner only amounted to about £360, and, in consequence of this, the chairman decided that he would keep his list open for some time longer.

